# Synthesis of the Tetrasaccharide Motif and Its Structural Analog Corresponding to the Lipopolysaccharide of *Escherichia coli* O75

**DOI:** 10.1371/journal.pone.0037291

**Published:** 2012-05-25

**Authors:** Abhijit Sau, Anup Kumar Misra

**Affiliations:** Bose Institute, Molecular Medicine Division, Kolkata, India; National Cancer Institute at Frederick, United States of America

## Abstract

**Background:**

Extraintestinal pathogenic *E. coli* are mostly responsible for a diverse spectrum of invasive human and animal infections leading to the urinary tract infections. Bacterial lipopolysaccharides are responsible for their pathogenicity and their interactions with host immune responses. In spite of several breakthroughs in the development of therapeutics to combat urinary tract infections and related diseases, the emergence of multidrug-resistant bacterial strains is a serious concern. Lipopolysaccharides are attractive targets for the development of long-term therapeutic agents to eradicate the infections. Since the natural sources cannot provide the required amount of oligosaccharides, development of chemical synthetic strategies for their synthesis is relevant to gain access to a reservoir of oligosaccharides and their close analogs.

**Methodology:**

Two tetrasaccharide derivatives were synthesized from a single disaccharide intermediate. β-d-mannoside moiety was prepared from β-d-glucoside moiety following oxidation–reduction methodology. A [2+2] stereoselective block glycosylation strategy has been adopted for the preparation of tetrasaccharide derivative. α-d-Glucosamine moiety was prepared from α-d-mannosidic moiety following triflate formation at C-2 and S_N_^2^ substitution. A one-pot iterative glycosylation exploiting the orthogonal property of thioglycoside was carried out during the synthesis of tetrasaccharide analog.

**Results:**

Synthesis of the tetrasaccharide motif (**1**) and its structural analog (**2**) corresponding to the lipopolysaccharide of *Escherichia coli* O75 was successfully achieved in excellent yield. Most of the reactions are clean and high yielding. Both compounds **1** and **2** were synthesized as their 4-methoxyphenyl glycoside, which can act as a temporary anomeric protecting group for further use of these tetrasaccharides in the preparation of glycoconjugates.

## Introduction

**Figure 1:**
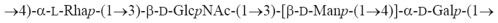
Tetrasaccharide repeating unit corresponding to the *O*-specific lipopolysaccharide of *Escherichia coli* O75.

*Escherichia coli* (*E. coli*) are opportunistic pathogen belong to the Gram-negative Enterobacteriaceae. Pathogenic *E. coli* are the major causative agents for a number of extra intestinal infections in humans and animals [1]. One of the most frequently occurring *E. coli* infections is urinary tract infections. *Escherichia coli* strains are responsible for 60 to 80% of community acquired urinary tract infections in children and adults [2], [3]. Extra intestinal pathogenic *E. coli* are mostly responsible for a diverse spectrum of invasive human and animal infections leading to pyelonephritis in the developing and developed countries [4], [5], [6]. The mechanism of such kind of ascending urinary tract infections has been explained based on the interactions between *E. coli* adhesion and their uroepithelial receptor ligands [7]. The most of the urinary tract infections found in human are caused by a small number of *E. coli* O-serogroups e.g. O4, O6, O14, O22, O75 and O83 [8]. Furthermore, they have phenotypes that are epidemiologically associated with cystitis and acute pyelonephritis in the normal urinary tract [9], [10]. In this context, a revised structure of the *E. coli* O75 lipopolysaccharide has been reported by Erbing *et. al.* [11] (Figure 1). Bacterial lipopolysaccharides play vital roles for their pathogenicity and their interactions with host immune responses.

**Figure 2:**
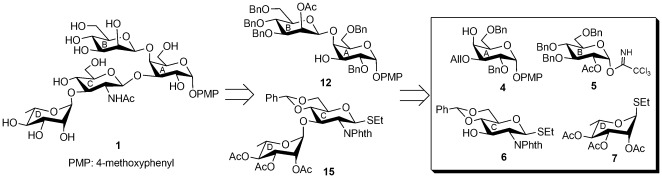
Structure of the tetrasaccharide repeating unit corresponding to the *O*-specific lipopolysaccharide of *Escherichia coli* O75.

**Figure 3:**
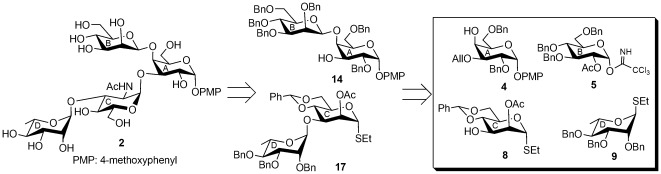
Structure of the tetrasaccharide analog related to the repeating unit of the *O*-specific lipopolysaccharide of *Escherichia coli* O75.

In spite of several breakthroughs in the development of therapeutics to combat urinary tract infections and related diseases, the emergence of multi drug resistant bacterial strains is a serious concern. The epidemiological data for the urinary tract infections caused by multi-drug resistant *E. coli* O75 and other strains in the developed and developing countries have been well documented [12]–[14]. Bacterial lipopolysaccharides and their fragments have been used to prepare several glycoconjugate derivatives towards the development of long term therapeutic agents to eradicate the infections [15]–[17]. In order to establish a clear understanding on the biological potential of the lipooligosaccharide of a particular strain and its glycoconjugates, it is essential to carry out several biological experiments which require pure oligosaccharide in large quantity. Since the natural sources can not provide the required amount of the oligosaccharides, development of chemical synthetic strategies for their synthesis are relevant to get access to a reservoir of oligosaccharides and their close analogs. As a part of the ongoing studies on the synthesis of oligosaccharides of bacterial origin for their use in the preparation of glycoconjugate derivatives, concise chemical synthetic strategies for the synthesis of tetrasaccharide repeating unit (**1**) (Figure 2) corresponding to the lipopolysaccharide of *Escherichia coli* O75 and its close tetrasaccharide analog (**2**) (Figure 3) are reported herein. The difference between tetrasaccharides **1** and **2** is that the d-glucosamine moiety is 1,2-*trans* linked in compound **1** whereas it is 1,2-*cis* linked in compound **2**. Both tetrasaccharides **1** and **2** were synthesized as their 4-methoxyphenyl (PMP) glycosides.

## Results and Discussion

The synthesis of the tetrasaccharide **1** and its close structural analog **2** as their 4-methoxyphenyl glycosides was achieved by series of stereoselective glycosylations of a number of suitably functionalized monosaccharide derivatives **4**, **5** [18], **6**, **7**, **8** [19], and **9** [20] prepared from the commercially available reducing sugars using synthetic methodologies reported earlier. Since, the preparation of β-linked d-mannose moiety from d-mannose and α-d-glucosamine moiety from d-glucosamine are challenging issues, these two moieties are successfully prepared using β-d-glucosyl moiety and α-d-mannosyl moiety respectively as the precursors after completion of the glycosylations with required stereochemical outcome. The key features of this synthetic strategy include, (a) use of a common disaccharide derivative **11** for the preparation of both **1** and **2**; (b) convenient conversion of β-d-glucoside moiety to β-d-mannoside moiety using Dess-Martin periodinane oxidation of C-2 followed by sodium borohydride reduction of the keto- group; (c) [2+2] stereoselective block glycosylation; (d) use of α-d-mannosidic moiety as a precursor for α-d-glucosamine moiety; (e) triflate formation followed by S_N_^2^ substitution by azido group at C-2 position of α-d-mannosidic moiety; (f) high yield in most of the intermediate steps.

4-Methoxyphenyl 3-*O*-allyl-2,6-di-*O*-benzyl-α-d-galactopyranoside (**4**) was prepared from 4-methoxyphenyl 2,3,4,6-tetra-*O*-acetyl-α-d-galactopyranoside (**3**) [21] using a series of reactions involving (a) deacetylation using sodium methoxide; (b) 3,4-*O*-isopropylidene ketal formation using 2,2-dimethoxypropane and *p*-toluenesulfonic acid [22]; (c) benzylation using benzyl bromide and sodium hydroxide [23]; (d) acidic hydrolysis of isopropylidene ketal and (e) selective 3-*O*-allylation via stannylidene acetal formation [24] in 70% overall yield (Scheme S1).

Stereo selective glycosylation of compound **4** with glucosyl trichloroacetimidate derivative **5** [18] in the presence of trifluoromethane sulfonic acid (TfOH) [25] furnished 4-methoxyphenyl (2-*O*-acetyl-3,4,6-tri-*O*-benzyl-β-d-glucopyranosyl)-(1→4)-3-*O*-allyl-2,6-di-*O*-benzyl-α-d-galactopyranoside (**10**) in 72% yield. Formation of compound **10** was confirmed from its spectral analysis [δ 5.24 (d, *J* = 3.5 Hz, H-1_A_), 4.61 (d, *J* = 8.0 Hz, H-1_B_) in the ^1^H NMR and δ 101.8 (C-1_B_), 97.4 (C-1_A_) in the ^13^C NMR spectra]. The coupling constant value (*J*_1,2_ = 8.0 Hz) unambiguously confirmed the β-linkage of the d-glucose moiety in compound **10**. The d-glucopyranosyl moiety in compound **10** was converted into a d-mannopyranosyl moiety by epimerization at C-2. For this purpose, removal of acetyl group in compound **10** using sodium methoxide and oxidation of the hydroxyl group using Dess-Martin Periodinane [26] followed by reduction of the resulting keto group using sodium borohydride [27] resulted in the β-d-mannopyranosyl moiety, which was acetylated to give compound **11** in 76% overall yield. The spectral analysis of compound **11** supported its formation [δ 5.19 (d, *J* = 3.0 Hz, H-1_A_), 4.81 (br s, H-1_B_) in the ^1^H NMR and δ 99.1 (C-1_B_), 97.8 (C-1_A_) in the ^13^C NMR spectra]. The formation of β-d-mannosidic residue in compound **11** was unambiguously confirmed from the NMR spectral data [δ 4.81 (br s, H-1_B_) and δ 99.1 (C-1_B_) in the ^1^H NMR and ^13^C NMR spectra respectively]. Since the presence of β-d-glucosidic moiety in compound **10** was unambiguously confirmed from the NMR spectra and the β-d-mannosidic moiety of the compound **11** was prepared from the β-d-glucosidic moiety of compound **10** by oxidation-reduction at the C-2 center without affecting the stereochemistry at the glycosyl linkages, the glycosyl linkage of the d-mannosidic moiety in compound **11** remained as β-linked (1,2-*cis*). Compound **11** was treated with palladium chloride [28] to remove allyl group to give compound **12** in 76% yield. In another set of experiment, the *O*-acetyl group of compound **11** was transformed into benzyl group on treatment with benzyl bromide in the presence of solid sodium hydroxide [23] to give compound **13** in 90% yield, which was treated with palladium chloride [28] to furnish compound **14** in 78% yield. Spectral analysis of compound **14** supported its formation [δ 5.46 (d, *J* = 3.2 Hz, H-1_A_), 4.77 (br s, H-1_B_) in ^1^H NMR and δ 101.8 (C-1_B_), 96.8 (C-1_A_) in the ^13^C NMR spectra] (Scheme S2).

Stereoselective glycosylation of thioglycoside derivative **6** with thioglycoside derivative **7** in the presence of a combination of *N*-iodosuccinimide (NIS) and trifluoromethane sulfonic acid (TfOH) [29], [30] furnished disaccharide thioglycoside derivative **15** [31] in 77% yield exploiting the concept of “Relative reactivity values” of thioglycosides discussed in the earlier report [31] (Scheme S3).

Using block synthetic strategy, stereoselective glycosylation of disaccharide thioglycoside **15** with disaccharide acceptor **12** in the presence of a combination of NIS and TfOH [29], [30] furnished tetrasaccharide derivative **16** in 72% yield. Spectral analysis of compound **16** confirmed its formation [δ 5.49 (s, PhC*H*), 5.43 (d, *J* = 8.0 Hz, H-1_C_), 4.90 (br s, H-1_B_), 4.66 (d, *J* = 3.5 Hz, H-1_A_) in the ^1^H NMR and δ 102.1 (Ph*C*H), 99.5 (C-1_C_), 98.8 (C-1_B_), 97.3 (2 C, C-1_A_, C-1_D_) in the ^13^C NMR spectra]. Compound **16** was subjected to a series of reactions involving (a) transformation of *N*-phthalimido group to acetamido group by hydrazinolysis [31] followed by *N*-acetylation; (b) removal of benzyl groups and benzylidene acetal by exhaustive hydrogenolysis over pearlman's catalyst [32] and finally, (c) saponification using sodium methoxide to furnish target tetrasaccharide **1** in 57% overall yield. Spectral analysis of compound **1** unambiguously supported its formation [ δ 5.30 (d, *J* = 8.5 Hz, H-1_C_), 5.11 (d, *J* = 3.5 Hz, H-1_A_), 4.80 (br s, H-1_D_), 4.46 (br s, H-1_B_) in the ^1^H NMR and δ 100.6 (C-1_B_), 100.2 (C-1_D_), 99.0 (C-1_C_), 98.4 (C-1_A_) in the ^13^C NMR spectra] (Scheme S4).

In another synthetic strategy for the synthesis of compound **2**, a tetrasaccharide derivative **18** was prepared using a convergent reaction protocol, which involves iodonium ion promoted iterative stereoselective glycosylations in one-pot. Stereoselective condensation of thioglycoside **8** and thioglycoside **9** in the first step of the iterative glycosylation using NIS-TfOH [29], [30] as promoter furnished disaccharide thioglycoside derivative **17** following the principle of “armed-disarmed” glycosylation concept [33], [34]. Immediate reaction of the *in situ* generated disaccharide thioglycoside derivative **17** with the disaccharide acceptor **14** in the presence of same activator present in the reaction pot led to the formation of tetrasaccharide **18** in 71% overall yield together with a minor quantity (∼8%) of other isomeric product generated from the first step of glycosylation, which was separated by column chromatography. In the first step of the iterative glycosylation, although both compound **8** and **9** are thioethyl glycosides, presence of electron donating benzyl group at C-2 makes compound **9** activated or armed to act as glycosyl donor, whereas compound **8** acted as glycosyl acceptor because of the deactivation due to the presence of electron withdrawing *O*-acetyl group at C-2. In the second step, required 1,2-*trans* glycosylated product was obtained due to the presence of *O*-acetyl group at C-2 of the *in situ* formed disaccharide donor **17**. The formation of tetrasaccharide derivative **18** was unambiguously confirmed from its spectral analysis [δ 5.58 (s, PhC*H*), 5.41 (d, *J* = 3.0 Hz, H-1_A_), 5.04 (br s, H-1_D_), 5.02 (br s, H-1_C_), 4.63 (br s, 1 H, H-1_B_) in the ^1^H NMR and δ 101.5 (C-1_B_), 101.1 (Ph*C*H), 97.6 (C-1_A_), 95.0 (C-1_D_), 94.0 (C-1_C_) in the ^13^C NMR spectra]. The stereochemistry of the anomeric centers were further confirmed from the *J*_C-1/H-1_ values in the proton coupled ^13^C NMR spectrum [Appearance *J*_C-1/H-1_: 172.0 Hz (α-Rhap), 174.0 (α-Manp), 168.0 Hz (α-Galp), 156.0 Hz (β-Manp)] [35], [36]. The D-mannose moiety in compound **18** was converted to the d-glucosamine moiety following a series of reactions involving (a) removal of *O*-acetyl group using sodium methoxide; (b) treatment of the resulting hydroxyl group with triflic anhydride to form triflate derivative; (c) treatment of the triflate derivative with sodium azide to substitute triflate group with azido group by S_N_^2^ substitution [37]. Finally, removal of the benzyl groups and transformation of the azido group to acetamido group by hydrogenolysis followed by *N*-acetylation furnished compound **2** in 51% overall yield. Spectral analysis of compound **2** unambiguously supported its formation [δ 5.38 (d, *J* = 3.5 Hz, H-1_A_), 5.10 (d, *J* = 1.5 Hz, H-1_C_), 4.90 (d, *J* = 1.5 Hz, H-1_D_), 4.61 (br s, H-1_B_) in the ^1^H NMR and δ 102.6 (C-1_B_), 100.7 (C-1_A_), 99.6 (C-1_C_), 98.0 (C-1_D_) in the ^13^C NMR spectra] (Scheme S5).

In summary, synthesis of the tetrasaccharide motif (**1**) and its structural analog (**2**) corresponding to the *O*-specific lipopolysaccharide of *Escherichia coli* O75 was successfully achieved in excellent yield. A number of notable features are present in the synthetic strategies, which include (a) preparation of β-d-mannosyl moiety from β-d-glucosyl moiety using oxidation-stereoselective reduction approach; (b) preparation of α-d-glucosaminyl moiety from α-d-mannosyl moiety by S_N_^2^ substitution of triflate with azido group; (c) one-pot two iterative glycosylations; (d) [2+2] block glycosylation; (e) exploitation of orthogonal property of thioglycosides. Most of the reactions are clean and high yielding.

## Materials and Methods

### General methods

All reactions were monitored by thin layer chromatography over silica gel coated TLC plates. The spots on TLC were visualized by warming ceric sulphate (2% Ce(SO_4_)_2_ in 2N H_2_SO_4_) sprayed plates in hot plate. Silica gel 230–400 mesh was used for column chromatography. 1D and 2D NMR spectra were recorded on Bruker Avance 500 and 600 MHz spectrometer using CDCl_3_ and CD_3_OD as solvents and TMS as internal reference unless stated otherwise. Chemical shift value is expressed in δ ppm. ESI-MS were recorded on a Micromass mass spectrometer. Elementary analysis was carried out on Carlo Erba analyzer. Optical rotations were measured at 25 °C on a Jasco-P 2000 polarimeter. Commercially available organic solvents of adequate purity are used in all reactions.

### 4-Methoxyphenyl 3-*O*-allyl-2,6-di-*O*-benzyl-α-d-galactopyranoside (4)

A solution of compound **3** (5.0 g, 11.0 mmol) in 0.1 M CH_3_ONa in CH_3_OH (50 mL) was stirred at room temperature for 3 h and neutralized with Amberlite IR 120 (H^+^) resin. The reaction mixture was filtered and concentrated under reduced pressure. To a solution of the crude product in anhydrous DMF (15 mL) were added 2,2-dimethoxypropane (3 mL, 24.4 mmol) and *p*-TsOH (250.0 mg) and reaction mixture was allowed to stir at room temperature for 12 h. The reaction mixture was cooled to 0 °C and powdered NaOH (2.0 g, 50.0 mmol) was added to it followed by benzyl bromide (2.6 mL, 21.9 mmol) and the reaction was stirred at room temperature for 4 h. The reaction mixture was diluted with water (150 mL) and the extracted with EtOAc (2×100 mL). The organic layer was washed with water, dried (Na_2_SO_4_) and concentrated. A solution of the crude benzylated product in 80% AcOH (100 mL) was stirred at 80 °C for 1.5 h. The solvents were evaporated under reduced pressure and co-evaporated with toluene and the crude product was passed through a short pad of SiO_2_. To a solution of the dihydroxyl compound in anhydrous CH_3_OH (150 mL) was added Bu_2_SnO (4.0 g, 16.07 mmol) and the reaction was allowed to stir at 70 °C for 3 h. The solvents were removed under reduced pressure and the stannylidene acetal was dissolved in dry DMF (10 mL). To the solution of the crude product were added allyl bromide (1.4 mL, 16.2 mmol) and Bu_4_NBr (500 mg) and the reaction mixture was allowed to stir at 60 °C for 8 h. The reaction mixture was diluted with water and extracted with EtOAc (2×100 mL). The organic layer was washed with 1 N HCl, satd. NaHCO_3_ and water in succession, dried (Na_2_SO_4_) and concentrated. The crude product was purified over SiO_2_ using hexane-EtOAc (4∶1) as eluant to give pure compound **4** (3.9 g, 70%). Yellow oil; [α]_D_^25^+110.5 (*c* 1.2, CHCl_3_); IR (neat): 3475, 2928, 1504, 1216, 1097, 1040, 756, 698 cm^−1^; ^1^H NMR (CDCl_3_, 500 MHz): δ 7.34-7.23 (m, 10 H, Ar-H), 7.03 (d, *J* = 9.0 Hz, 2 H, Ar-H), 6.77 (d, *J* = 9.0 Hz, 2 H, Ar-H), 6.00-5.93 (m, 1 H, C*H* = CH_2_), 5.35-5.31 (m, 1 H, CH = C*H*_2_), 5.33 (d, *J* = 3.0 Hz, 1 H, H-1), 5.21-5.19 (m, 1 H, CH = C*H*_2_), 4.80 (d, *J* = 12.0 Hz, 1 H, PhC*H*_2_), 4.67 (d, *J* = 12.0 Hz, 1 H, PhC*H*_2_), 4.49 (s, 2 H, PhC*H*_2_), 4.29-4.25 (m, 2 H, H-5, O-C*H*_2_-), 4.15-4.12 (m, 2 H, H-4, O-C*H*_2_), 3.94-3.93 (m, 2 H, H-6_ab_), 3.77 (dd, *J* = 10.0, 5.4 Hz, 1 H, H-2), 3.73 (s, 3 H, OC*H*_3_), 3.66 (dd, *J* = 10.0, 6.1 Hz, 1 H, H-3); ESI-MS: 529.2 [M+Na]^+^; Anal. Calcd. for C_30_H_34_O_7_ (506.23): C, 71.13; H, 6.76%; found: 70.90; H, 7.00%.

### 4-Methoxyphenyl (2-*O*-acetyl-3,4,6-tri-*O*-benzyl-β-d-glucopyranosyl)-(1→4)-3-*O*-allyl-2,6-di-*O*-benzyl-α-d-galactopyranoside (10)

A solution of compound **4** (2.0 g, 3.95 mmol) and compound **5** (3.0 g, 4.71 mmol) in anhydrous CH_2_Cl_2_ (25 mL) was cooled to −25°C. To the cooled reaction mixture was added TfOH (100 µL) and the reaction mixture was allowed to stir at same temperature for 1 h. The reaction mixture was diluted with CH_2_Cl_2_ (150 mL) and successively washed with satd. NaHCO_3_ and water, dried (Na_2_SO_4_) and concentrated under reduced pressure. The crude product was purified over SiO_2_ using hexane-EtOAc (4∶1) as eluant to give pure compound **10** (2.8 g, 72%). Colorless oil; [α]_D_^25^+38.8 (*c* 1.2, CHCl_3_); IR (neat): 2823, 2260, 1542, 1344, 1152, 1213, 1104, 1070, 737, 567 cm^−1^; ^1^H NMR (CDCl_3_, 500 MHz): δ 7.28-7.10 (m, 25 H, Ar-H), 6.95 (d, *J* = 9.0 Hz, 2 H, Ar-H), 6.67 (d, *J* = 9.0 Hz, 2 H, Ar-H), 5.91-5.80 (m, 1 H, C*H* = CH_2_), 5.32-5.28 (m, 1 H, CH = C*H*_2_), 5.24 (d, *J* = 3.5 Hz, 1 H, H-1_A_), 5.11-5.08 (m, 1 H, CH = C*H*_2_), 4.94 (t, *J* = 9.5 Hz each, 1 H, H-2_B_), 4.74-4.63 (4 d, *J* = 11.8 Hz each, 4 H, PhC*H*_2_), 4.61 (d, *J* = 8.0 Hz, 1 H, H-1_B_), 4.56 (d, *J* = 11.5 Hz, 1 H, PhC*H*_2_), 4.51 (d, *J* = 11.5 Hz, 1 H, PhC*H*_2_), 4.45 (d, *J* = 12.0 Hz, 1 H, PhC*H*_2_), 4.38 (d, *J* = 12.0 Hz, 1 H, PhC*H*_2_), 4.35 (d, *J* = 12.0 Hz, 1 H, PhC*H*_2_), 4.33 (d, *J* = 12.0 Hz, 1 H, PhC*H*_2_), 4.27-4.10 (m, 2 H, O-C*H*_2_), 4.08-4.06 (m, 2 H, H-4_A_, H-5_A_), 3.84 (dd, *J* = 10.0, 2.5 Hz, 1 H, H-2_A_), 3.80 (dd, *J* = 10.0, 3.5 Hz, 1 H, H-3_A_), 3.77-3.74 (m, 1 H, H-6_aB_), 3.68 (dd, *J* = 9.5, 3.5 Hz, 1 H, H-3_B_), 3.67 (s, 3 H, OC*H*_3_), 3.65-3.56 (m, 4 H, H-4_B_, H-6_bB_, H-6_abA_), 3.39-3.37 (m, 1 H, H-5_B_), 1.92 (s, 3 H, OC*H*_3_); ^13^C NMR (CDCl_3_, 125 MHz): δ 169.8 (*C*OCH_3_), 155.1-114.4 (Ar-C, *C*H = *C*H_2_), 101.8 (C-1_B_), 97.4 (C-1_A_), 83.0 (C-3_B_), 77.8 (C-4_B_), 77.6 (C-5_B_), 76.7 (C-4_A_), 75.3 (C-2_A_), 75.1 (Ph*C*H_2_), 75.0 (Ph*C*H_2_), 74.8 (C-3_A_), 73.8 (C-2_B_), 73.5 (Ph*C*H_2_), 73.4 (Ph*C*H_2_), 73.0 (Ph*C*H_2_), 71.9 (O-*C*H_2_), 70.2 (C-5_B_), 71.1 (C-6_B_), 69.0 (C-6_A_), 55.6 (O*C*H_3_), 21.2 (CO*C*H_3_); ESI-MS: 1003.4 [M+Na]^+^; Anal. Calcd. for C_59_H_64_O_13_ (980.43): C, 72.23; H, 6.57%; found: 72.00; H, 6.85%.

### 4-Methoxyphenyl (2-*O*-acetyl-3,4,6-tri-*O*-benzyl-β-d-mannopyranosyl)-(1→4)-3-*O*-allyl-2,6-di-*O*-benzyl-α-d-galactopyranoside (11)

A solution of compound **10** (2.5 g, 2.55 mmol) in 0.1 M CH_3_ONa in CH_3_OH (25 mL) was stirred at room temperature for 2 h and neutralized with Amberlite IR 120 (H^+^) resin. The reaction mixture was filtered and concentrated. To a solution of the deacetylated product in anhydrous CH_2_Cl_2_ (15 mL) was added Dess-Martin Periodinane (2.0 g, 4.72 mmol) and the reaction mixture was allowed to stir at room temperature for 1 h. The reaction mixture was diluted with CH_2_Cl_2_ (100 mL) and the organic layer was successively washed with 5% Na_2_S_2_O_3_, satd. NaHCO_3_ and water, dried (Na_2_SO_4_) and concentrated under reduced pressure. To a solution of the crude keto product in CH_3_OH (50 mL) was added NaBH_4_ (1.5 g, 39.65 mmol) and the reaction mixture was allowed to stir at room temperature for 12 h. The solvents were removed under reduced pressure and the crude mass was dissolved in CH_2_Cl_2_ (100 mL). The organic layer was successively washed with 1 N HCl, satd. NaHCO_3_ and water, dried (Na_2_SO_4_) and evaporated to dryness. A solution of the crude epimerized product in acetic anhydride-pyridine (10 mL, 1∶1 v/v) was kept at room temperature for 2 h. The solvents were removed under reduced pressure and the crude product was purified over SiO_2_ using hexane-EtOAc (5∶1) as eluant to give pure compound **11** (1.9 g, 76%). Yellow oil; [α]_D_^25^+27.6 (*c* 1.2, CHCl_3_); IR (neat): 2818, 2220, 1549, 1365, 1159, 1235, 1100, 739 cm^−1^; ^1^H NMR (CDCl_3_, 125 MHz): δ 7.33-7.11 (m, 25 H, Ar-H), 6.94 (d, *J* = 9.0 Hz, 2 H, Ar-H), 6.66 (d, *J* = 9.0 Hz, 2 H, Ar-H), 5.84-5.78 (m, 1 H, C*H* = CH_2_), 5.72 (d, *J* = 2.5 Hz, 1 H, H-2_B_), 5.23-5.20 (m, 1 H, CH = C*H*_2_), 5.19 (d, *J* = 3.0 Hz, 1 H, H-1_A_), 5.05-5.03 (m, 1 H, CH = C*H*_2_), 4.81 (br s, 1 H, H-1_B_), 4.78-4.31 (10 d, *J* = 11.8 Hz each, 10 H, PhC*H*_2_), 4.26-4.23 (m, 1 H, O-C*H*_2_), 4.19 (br s, 1 H, H-4_A_), 4.10-4.06 (m, 2 H, H-5_A_, O-C*H*_2_), 3.93 (dd, *J* = 10.0, 3.5 Hz, 1 H, H-2_A_), 3.88 (dd, *J* = 10.0, 2.5 Hz, 1 H, H-3_A_), 3.71-3.63 (m, 4 H, H 4_B_, H-6_abA_, H-6_aB_), 3.64 (s, 3 H, OC*H*_3_), 3.58-3.53 (m, 2 H, H-3_B_, H-6_bB_), 3.37-3.34 (m, 1 H, H-5_B_), 2.09 (s, 3 H, COC*H*_3_); ^13^C NMR (CDCl_3_, 125 MHz): δ 170.6 (*C*OCH_3_), 155.1-114.4 (Ar-C, *C*H = *C*H_2_), 99.1 (C-1_B_), 97.8 (C-1_A_), 80.4 (C-3_B_), 77.9 (C-3_A_), 76.4 (C-2_A_), 75.6 (C-5_B_), 75.2 (Ph*C*H_2_), 74.4 (C-4_B_), 74.2 (C-4_A_), 73.7 (Ph*C*H_2_), 73.5 (Ph*C*H_2_), 73.0 (Ph*C*H_2_), 71.9 (Ph*C*H_2_), 71.4 (Ph*C*H_2_), 70.0 (C-5_A_), 69.9 (C-6_B_), 69.4 (C-6_A_), 68.1 (C-2_B_), 55.6 (O*C*H_3_), 21.1 (CO*C*H_3_); ESI-MS: 1003.4 [M+Na]^+^; Anal. Calcd. for C_59_H_64_O_13_ (980.43): C, 72.23; H, 6.57%; found: 72.04; H, 6.80%.

### 4-Methoxyphenyl (2-*O*-acetyl-3,4,6-tri-*O*-benzyl-β-d-mannopyranosyl)-(1→4)-2,6-di-*O*-benzyl-α-d-galactopyranoside (12)

To a solution of compound **11** (900.0 mg, 0.92 mmol) in anhydrous CH_3_OH (5 mL) was added PdCl_2_ (100.0 mg, 0.56 mmol) and the reaction mixture was allowed to stir at room temperature for 1 h. The solvents were removed under reduced pressure and the crude product was purified over SiO_2_ using hexane-EtOAc (4∶1) to give pure compound **12** (660.0 mg, 76%). Yellow oil; [α]_D_^25^+40 (*c* 1.2, CHCl_3_); IR (neat): 2834, 2297, 1534, 1371, 1156, 1218, 1080, 736 cm^−1^; ^1^H NMR (CDCl_3_, 500 MHz): δ 7.35-7.0 (m, 25 H, Ar-H), 6.91 (d, *J* = 9.0 Hz, 2 H, Ar-H), 6.67 (d, *J* = 9.0 Hz, 2 H, Ar-H), 5.62 (d, *J* = 3.0 Hz, 1 H, H-2_B_), 5.21 (d, *J* = 3.5 Hz, 1 H, H-1_A_), 4.78 (d, *J* = 10.5 Hz, 1 H, PhC*H*_2_), 4.69 (br s, 1 H, H-1_B_), 4.67-4.61 (3 d, *J* = 11.0 Hz each, 3 H, PhC*H*_2_), 4.50-4.23 (6 d, *J* = 11.0 Hz each, 6 H, PhC*H*_2_), 4.13-4.11 (m, 2 H, H-4_A_, H-5_A_), 4.09-4.06 (m, 1 H, H-6_aA_), 3.76-3.68 (m, 4 H, H-2_A_, H-4_B_, H-6_aB_, H-6_bA_), 3.67 (s, 3 H, OC*H*_3_), 3.65-3.61 (m, 1 H, H-3_B_), 3.59 (dd, *J* = 10.0, 2.6 Hz, 1 H, H-3_A_), 3.54-3.51 (m, 1 H, H-6_bB_), 3.34-3.31 (m, 1 H, H-5_B_), 2.10 (s, 3 H, COC*H*_3_); ^13^C NMR (CDCl_3_, 125 MHz): δ 171.5 (*C*OCH_3_), 155.1-114.5 (Ar-C), 100.5 (C-1_B_), 96.9 (C-1_A_), 80.1 (C-3_B_), 78.5 (C-3_A_), 76.6 (C-2_A_), 75.5 (C-5_B_), 75.2 (Ph*C*H_2_), 74.3 (C-4_B_), 73.5 (C-4_A_), 73.0 (Ph*C*H_2_), 72.8 (Ph*C*H_2_), 71.5 (Ph*C*H_2_), 69.8 (Ph*C*H_2_), 69.5 (C-5_A_), 69.4 (C-6_B_), 69.3 (C-6_A_), 68.7 (C-2_B_), 55.6 (O*C*H_3_), 21.3 (CO*C*H_3_); ESI-MS: 963.4 [M+Na]^+^; Anal. Calcd. for C_56_H_60_O_13_ (940.40): C, 71.47; H, 6.43%; found: 71.26; H, 6.60%.

### 4-Methoxyphenyl (2,3,4,6-tetra-*O*-benzyl-β-d-mannopyranosyl)-(1→4)-3-*O*-allyl-2,6-di-*O*-benzyl-α-d-galactopyranoside (13)

To a solution of compound **11** (900.0 mg, 0.92 mmol) in THF (5 mL) were added powdered NaOH (0.2 g, 5.0 mmol), benzyl bromide (250 µL, 2.10 mmol) and Bu_4_NBr (25.0 mg) and reaction mixture was allowed to stir at room temperature for 3 h. The reaction mixture was diluted with CH_2_Cl_2_ (50 mL) and the organic layer was washed with water, dried (Na_2_SO_4_) and concentrated under reduced pressure. The crude product was purified over SiO_2_ using hexane-EtOAc (7∶1) as eluant to give pure compound **13** (850.0 mg, 90%). Yellow oil; [α]_D_^25^+20 (*c* 1.2, CHCl_3_); IR (neat): 3443, 2923, 2860, 1508, 1454, 1362, 1213, 1114, 1070, 1027, 737, 697 cm^−1^; ^1^H NMR (CDCl_3_, 500 MHz): δ.7.55-7.14 (m, 30 H, Ar-H), 7.10 (d, *J* = 9.0 Hz, 2 H, Ar-H), 6.75 (d, *J* = 9.0 Hz, 2 H, Ar-H), 5.90-5.84 (m, 1 H, C*H* = CH_2_), 5.40 (d, *J* = 3.0 Hz, 1 H, H-1_A_), 5.25-5.09 (m, 2 H, C*H* = CH_2_), 4.93 (d, *J* = 12.0 Hz, 1 H, PhC*H*_2_), 4.89 (d, *J* = 12.0 Hz, 1 H, PhC*H*_2_), 4.79 (d, *J* = 12.0 Hz, 1 H, PhC*H*_2_), 4.76 (br s, 1 H, H-1_B_), 4.74 (d, *J* = 12.0 Hz, 1 H, PhC*H*_2_), 4.68 (d, *J* = 12.0 Hz, 1 H, PhC*H*_2_), 4.61 (d, *J* = 12.0 Hz, 1 H, PhC*H*_2_), 4.54 (d, *J* = 12.0 Hz, 1 H, PhC*H*_2_), 4.52 (d, *J* = 12.0 Hz, 1 H, PhC*H*_2_), 4.48 (d, *J* = 12.0 Hz, 1 H, PhC*H*_2_), 4.39 (br s, 2 H, PhC*H*_2_), 4.30-4.28 (m, 1 H, OC*H*_2_-), 4.27 (br s, 1 H, H-4_A_), 4.25-4.23 (m, 1 H, H-5_A_), 4.15-4.12 (m, 1 H, OC*H*_2_-), 4.01 (d, *J* = 2.4 Hz, 1 H, H-2_B_), 3.97 (dd, *J* = 10.2, 3.0 Hz, 1 H, H-3_B_), 3.93-3.88 (m, 2 H, H-2_A_, H-4_B_), 3.85 (dd, *J* = 10.8, 4.2 Hz, 1 H, H-6_aB_), 3.79-3.75 (m, 3 H, H-6_bB_, H-6_abA_), 3.73 (s, 3 H, OC*H*_3_), 3.51 (dd, *J* = 9.6, 3.0 Hz, 1 H, H-3_B_), 3.42-3.40 (m, 1 H, H-5_B_); ^13^C NMR (CDCl_3_, 125 MHz): δ 155.6-114.4 (Ar-C, -*C*H = *C*H_2_), 101.6 (C-1_B_), 97.8 (C-1_A_), 82.5 (C-3_B_), 77.9 (C-3_A_), 76.0 (C-2_A_), 75.9 (C-5_B_), 75.2 (Ph*C*H_2_), 74.8 (C-4_B_), 73.9 (Ph*C*H_2_), 73.8 (C-2_B_), 73.7 (C-4_A_), 73.4 (Ph*C*H_2_), 73.1 (Ph*C*H_2_), 73.0 (Ph*C*H_2_), 71.7 (O*C*H_2_-), 71.4 (Ph*C*H_2_), 70.4 (C-6_B_), 70.3 (C-4_A_), 69.7 (C-6_A_), 55.5 (O*C*H_3_); ESI-MS: 1051.4 [M+Na]^+^; Anal. Calcd. for C_64_H_68_O_12_ (1028.47): C, 74.69; H, 6.66%; found: 74.90; H, 6.90%.

### 4-Methoxyphenyl (2,3,4,6-tetra-*O*-benzyl-β-d-mannopyranosyl)-(1→4)-2,6-di-*O*-benzyl-α-d-galactopyranoside (14)

To a solution of compound **13** (800.0 mg, 0.78 mmol) in anhydrous CH_3_OH (5 mL) was added PdCl_2_ (90.0 mg, 0.51 mmol) and the reaction mixture was allowed to stir at room temperature for 1 h. The solvents were removed under reduced pressure and crude product was purified over SiO_2_ using hexane-EtOAc (4∶1) to give pure compound **14** (600.0 mg, 78%). White solid; m.p. 132–133 °C (EtOH); [α]_D_^25^+38 (*c* 1.2, CHCl_3_); IR (KBr): 3440, 2936, 2854, 1512, 1466, 1345, 1209, 1121, 1078, 1037, 697 cm^−1^; ^1^H NMR (CDCl_3_, 500 MHz): δ 7.47-7.20 (m, 30 H, Ar-H), 7.08 (d, *J* = 9.0 Hz, 2 H, Ar-H), 6.77 (d, *J* = 9.0 Hz, 2 H, Ar-H), 5.46 (d, *J* = 3.2 Hz, 1 H, H-1_A_), 4.91-4.85 (3 d, *J* = 12.0 Hz each, 3 H, PhC*H*_2_), 4.77 (br s, 1 H, H-1_B_), 4.69 (d, *J* = 11.5 Hz, 1 H, PhC*H*_2_), 4.62-4.52 (m, 5 H, PhC*H*_2_), 4.47 (d, *J* = 11.5 Hz, 1 H, PhC*H*_2_), 4.43 (br s, 2 H, PhC*H*_2_), 4.25-4.22 (m, 2 H, H-3_A_, H-5_A_), 4.21 (br s, 1 H, H-4_A_), 3.97 (d, *J* = 2.6 Hz, 1 H, H-2_B_), 3.93-3.74 (m, 6 H, H-2_A_, H-4_B_, H-6_abA_, H-6_abB_), 3.73 (s, 3 H, OC*H*_3_), 3.54 (dd, *J* = 9.2, 2.7 Hz, 1 H, H-3_B_), 3.48-3.46 (m, 1 H, H-5_B_); ^13^C NMR (CDCl_3_, 125 MHz): δ 155.3-114.6 (Ar-C), 101.8 (C-1_B_), 96.8 (C-1_A_), 82.6 (C-3_B_), 75.8 (C-5_B_), 75.3 (C-2_A_), 75.0 (Ph*C*H_2_), 74.9 (Ph*C*H_2_), 74.0 (C-4_B_), 73.7 (C-3_A_), 73.5 (Ph*C*H_2_), 73.0 (Ph*C*H_2_), 72.5 (C-2_B_), 71.7 (Ph*C*H_2_), 70.7 (C-4_A_), 70.5 (C-5_A_), 69.8 (C-6_B_), 69.7 (C-6_A_), 55.6 (O*C*H_3_); ESI-MS: 1011.4 [M+Na]^+^; Anal. Calcd. for C_61_H_64_O_12_ (988.44): C, 74.07; H, 6.52%; found: 74.25; H, 6.75%.

### 4-Methoxyphenyl (2,3,4-tri-*O*-acetyl-α-l-rhamnopyranosyl)-(1→3)-(4,6-*O*-benzylidene-2-deoxy-2-*N*-phthalimido-β-d-glucopyranosyl)-(1→3)-[2-*O*-acetyl-3,4,6-tri-*O*-benzyl-β-d-mannopyranosyl-(1→4)]-2,6-di-*O*-benzyl-α-d-galactopyranoside (16)

To a solution of compound **12** (600.0 mg, 0.64 mmol) and compound **15** (550.0 mg, 0.77 mmol) in anhydrous CH_2_Cl_2_ (10 mL) was added MS 4 Å (1.5 g) and the reaction mixture was stirred under argon at room temperature for 30 min. The reaction mixture was cooled to −30 °C and NIS (210.0 mg, 0.93 mmol) and TfOH (2 µL) were added to it. After stirring at same temperature for 1 h the reaction mixture was filtered through a Celite® bed and washed with CH_2_Cl_2_ (50 mL). The organic layer was successively washed with 5% Na_2_S_2_O_3_, satd. NaHCO_3_ and water, dried (Na_2_SO_4_) and concentrated under reduced pressure. The crude product was purified over SiO_2_ using hexane-EtOAc (4∶1) as eluant to give pure compound **16** (730 mg, 72%). White solid; m.p. 98–100 °C (EtOH); [α]_D_^25^+10 (*c* 1.2, CHCl_3_); IR (KBr): 2929, 1745, 1454, 1373, 1233, 1100, 1069, 754, 698 cm^−1^; ^1^H NMR (CDCl_3_, 500 MHz): δ 7.76-6.99 (m, 34 H, Ar-H), 6.72 (d, *J* = 9.0 Hz, 2 H, Ar-H), 6.55 (d, *J* = 9.0 Hz, 2 H, Ar-H), 5.72-5.71 (m, 1 H, H-2_B_), 5.49 (s, 1 H, PhC*H*), 5.43 (d, *J* = 8.0 Hz, 1 H, H-1_C_), 5.15 (dd, *J* = 10.0, 3.5 Hz, 1 H, H-3_D_), 5.03 (d, *J* = 11.0 Hz, 1 H, PhC*H*_2_), 4.90 (br s, 1 H, H-1_B_), 4.86-4.83 (m, 2 H, PhC*H*_2_), 4.80 (t, *J* = 9.5 Hz each, 1 H, H-3_C_), 4.75 (t, *J* = 10.0 Hz each, 1 H, H-4_D_), 4.66 (d, *J* = 3.5 Hz, 1 H, H-1_A_), 4.64-4.63 (m, 1 H, H-2_D_), 4.53-4.50 (2 d, *J* = 11.0 Hz each, 2 H, PhC*H*_2_), 4.48 (br s, 1 H, H-1_D_), 4.40 (d, *J* = 11.0 Hz, 1 H, PhC*H*_2_), 4.32 (dd, *J* = 10.5, 2.0 Hz, 1 H, H-6_aC_), 4.28-4.21 (2 d, *J* = 12.0 Hz each, 2 H, PhC*H*_2_), 4.20-4.15 (m, 3 H, H-2_C_, PhC*H*_2_), 4.08-4.02 (m, 3 H, H-3_B_, H-5_A_, H-6_bC_), 3.96-3.90 (m, 1 H, H-5_D_), 3.75-3.63 (m, 7 H, H-4_A_, H-4_B_, H-5_C_, H-6_abA_, H-6_abB_), 3.62 (s, 3 H, OC*H*_3_), 3.61-3.58 (m, 1 H, H-4_C_), 3.56 (dd, *J* = 10.0, 2.5 Hz, 1 H, H-3_A_), 3.52-3.48 (m, 2 H, H-2_A_, H-5_B_), 1.97, 1.86, 1.80, 1.72 (4 s, 12 H, 4 COC*H*_3_), 0.52 (d, *J* = 6.0 Hz, 3 H, CC*H*_3_); ^13^C NMR (CDCl_3_, 125 MHz): δ 170.7, 169.9, 169.5 (4 *C*OCH_3_), 168.6, 167.8 (Phth*C*O), 155.1-114.3 (Ar-C), 102.1 (Ph*C*H), 99.5 (C-1_C_), 98.8 (C-1_B_), 97.3 (2 C, C-1_A_, C-1_D_), 80.6 (C-5_A_), 80.5 (C-5_C_), 76.6 (C-3_B_), 75.9 (C-3_A_), 75.4 (C-3_C_), 75.3 (C-2_A_), 75.0 (Ph*C*H_2_), 74.6 (C-5_B_), 73.8 (C-4_B_), 73.7 (Ph*C*H_2_), 73.6 (Ph*C*H_2_), 72.8 (Ph*C*H_2_), 71.8 (Ph*C*H_2_), 71.3 (C-4_D_), 70.4 (C-4_C_), 70.2 (C-6_B_), 69.8 (2 C, C-2_D_, C-6_C_), 68.7 (C-6_A_), 68.4 (C-3_D_), 68.3 (C-2_B_), 66.4 (C-4_A_), 66.2 (C-4_D_), 56.9 (C-2_C_), 55.5 (O*C*H_3_), 21.1, 20.8, 20.7, 20.6 (CO*C*H_3_), 16.5 (C*C*H_3_); ESI-MS: 1614.6 [M+Na]^+^; Anal. Calcd. for C_89_H_93_NO_26_ (1591.60): C, 67.12; H, 5.89%; found: 66.92; H, 6.12%.

### 4-Methoxyphenyl (2,3,4-tri-*O*-benzyl-α-l-rhamnopyranosyl)-(1→3)-(2-*O*-acetyl-4,6-*O*-benzylidene-α-d-mannopyranosyl)-(1→3)-[2,3,4,6-tetra-*O*-benzyl-β-d-mannopyranosyl-(1→4)]-2,6-di-*O*-benzyl-α-d-galactopyranoside (18)

To a solution of compound **8** (275.0 mg, 0.77 mmol) and compound **9** (375.0 mg, 0.78 mmol) in anhydrous CH_2_Cl_2_ (5 mL) was added MS 4 Å (1.0 g) and the reaction mixture was stirred under argon at room temperature for 30 min. The reaction mixture was cooled to −30 °C and NIS (190.0 mg, 0.84 mmol) and TfOH (5 µL) were added to it and the reaction was stirred at same temperature for 30 min. Thin layer chromatography (TLC; hexane-EtOAc, 5∶1) showed complete disappearance of the starting materials. To the reaction mixture were added compound **14** (650.0 mg, 0.66 mmol) and NIS (140 mg, 0.62 mmol) and the stirring reaction mixture was kept at same temperature for another 30 min. The reaction mixture was filtered through a Celite® bed and washed with CH_2_Cl_2_ (50 mL). The organic layer was successively washed with 5% Na_2_S_2_O_3_, satd. NaHCO_3_ and water, dried (Na_2_SO_4_) and concentrated under reduced pressure. The crude product was purified over SiO_2_ using hexane-EtOAc (4∶1) as eluant to give pure compound **18** (700.0 mg, 71%). White solid; m.p. 58–60 °C (EtOH); [α]_D_^25^+16 (*c* 1.2, CHCl_3_); IR (KBr): 2934, 1757, 1462, 1384, 1243, 1108, 1078, 757, 699 cm^−1^; ^1^H NMR (CDCl_3_, 500 MHz): δ 7.47-7.07 (m, 50 H, Ar-H), 6.77 (d, *J* = 9.0 Hz, Ar-H), 5.58 (s, 1 H, PhC*H*), 5.41 (d, *J* = 3.0 Hz, 1 H, H-1_A_), 5.28 (br s, 1 H, H-2_C_), 5.04 (br s, 1 H, H-1_D_), 5.02 (br s, 1 H, H-1_C_), 4.98-4.82 (m, 4 H, PhC*H*_2_), 4.71 (d, *J* = 12.0 Hz, PhC*H*_2_), 4.63 (br s, 1 H, H-1_B_), 4.62-4.46 (m, 11 H, PhCH_2_), 4.40-4.34 (m, 4 H, H-3_C_, H-5_C_, PhC*H*_2_), 4.32-4.28 (m, 3 H, H-4_A_, H-3_D_, H- 6_aC_), 4.26-4.22 (m, 1 H, H-5_A_), 4.13 (br s, 1 H, H-2_B_), 4.10-4.04 (m, 1 H, H-5_D_), 3.95-3.83 (m, H-2_A_, H-3_A_, H-4_B_, H-6_aB_, H-6_bC_), 3.82-3.76 (m, 4 H, H-4_C_, H-6_bB_, H-6_abA_), 3.75 (s, 3 H, OC*H*_3_), 3.70-3.66 (m, 1 H, H-3_B_), 3.63 (br s, 1 H, H-2_D_), 3.59-3.50 (m, 2 H, H-4_D_, H-5_B_), 2.06 (s, 3 H, COC*H*_3_), 1.07 (d, *J* = 5.8 Hz, 3 H, CC*H*_3_); ^13^C NMR (CDCl_3_, 125 MHz): δ 170.0 (*C*OCH_3_), 155.3-114.4 (Ar-C), 101.5 (C-1_B_), 101.1 (Ph*C*H), 97.6 (C-1_A_), 95.0 (C-1_D_), 94.0 (C-1_C_), 83.2 (C-2_D_), 80.4 (C-4_D_), 79.5 (C-4_C_), 76.5 (C-3_D_), 75.6 (C-5_B_), 74.9 (C-2_A_), 74.7 (C-3_A_), 74.6 (C-2_B_), 74.5 (C-3_B_), 74.4 (Ph*C*H_2_), 74.3 (2 C, C-4_B_, Ph*C*H_2_), 73.4 (Ph*C*H_2_), 73.0 (Ph*C*H_2_), 72.9 (2 C, 2 Ph*C*H_2_), 72.2 (Ph*C*H_2_), 71.9 (Ph*C*H_2_), 71.8 (Ph*C*H_2_), 71.5 (C-4_A_), 70.5 (C-3_C_), 70.1 (C-5_A_), 69.9 (C-6_A_), 68.8 (C-6_B_), 68.5 (C-6_C_), 68.0 (2 C, C-2_C_, C-5_D_), 63.9 (C-5_C_), 55.6 (O*C*H_3_); ESI-MS: 1719.7 [M+Na]^+^; Anal. Calcd. for C_103_H_108_O_22_ (1696.73): C, 72.86; H, 6.41%; found: 73.04; H, 6.65%.

### 4-Methoxyphenyl (α-l-rhamnopyranosyl)-(1→3)-(2-acetamido-2-deoxy-β-d-glucopyranosyl)-(1→3)-[β-d-mannopyranosyl-(1→4)]-α-d-galactopyranoside (1)

To a solution of compound **16** (700.0 mg, 0.44 mmol) in EtOH (30 mL) was added hydrazine monohydrate (0.3 mL, 6.18 mmol) and the reaction mixture was allowed to stir at 80 °C for 6 h. After removal of the solvents the crude mass was dissolved in acetic anhydride-pyridine (5 mL; 1∶1 v/v) and kept at room temperature for 1 h. The solvents were removed under reduced pressure to give the crude acetylated product. To a solution of the crude mass in CH_3_OH (15 mL) was added 20% Pd(OH)_2_-C (200.0 mg) and the reaction mixture was stirred at room temperature for 24 h. The reaction mixture was filtered through a Celite® bed and evaporated to dryness. A solution of the crude product in 0.1 M CH_3_ONa in CH_3_OH (15 mL) was allowed to stir at room temperature for 4 h and neutralized with Dowex 50W X8 (H^+^) resin. The reaction mixture was filtered and concentrated to dryness to give compound **1**, which was passed through a Sephadex® LH-20 column using CH_3_OH-H_2_O (4∶1) as eluant to give pure compound **1** (200.0 mg, 57%). Glass; [α]_D_^25^+46 (*c* 1.2, CH_3_OH); IR (KBr): 2929, 1763, 1458, 1392, 1257, 1100, 1082, 759, 697 cm^−1^; ^1^H NMR (CD_3_OD, 500 MHz): δ 6.85 (d, *J* = 9.0 Hz, 2 H, Ar-H), 6.77 (d, *J* = 9.0 Hz, 2 H, Ar-H), 5.30 (d, *J* = 8.5 Hz, 1 H, H-1_C_), 5.11 (d, *J* = 3.5 Hz, 1 H, H-1_A_), 4.80 (br s, 1 H, H-1_D_), 4.46 (br s, 1 H, H-1_B_), 4.33 (dd, *J* = 8.5 Hz each, 1 H, H-3_C_), 4.24 (br s, 1 H, H-2_D_), 4.06 (dd, *J* = 10.0, 2.5 Hz, H-3_D_), 3.93 (t, *J* = 8.5 Hz each, 1 H, H-2_C_), 3.48-3.80 (m, 1 H, H-6_aB_), 3.78-3.74 (m, 3 H, H-5_A_, H-5_D_, H-6_bB_), 3.66-3.56 (m, 2 H, H-4_A_, H-6_aA_), 3.60 (s, 3 H, OC*H*_3_), 3.55-3.51 (m, 4 H, H-2_A_, H-3_B_, H-6_ac_, H-6_bA_), 3.49-3.42 (m, 3 H, H-3_A_, H-5_C_, H-6_bC_), 3.40-3.36 (m, 2 H, H-2_B_, H-4_C_), 3.25-3.20 (m, 1 H, H-5_B_), 3.71 (t, *J* = 9.0 Hz each, 1 H, H-4_D_), 2.04 (s, 3 H, COC*H*_3_), 1.02 (d, *J* = 6.0 Hz, 3 H, CC*H*_3_); ^13^C NMR (CD_3_OD, 125 MHz): δ 173.0 (*C*OCH_3_), 154.6-114.9 (Ar-C), 100.6 (C-1_B_), 100.2 (C-1_D_), 99.0 (C-1_C_), 98.4 (C-1_A_), 77.9 (C-2_C_), 77.0 (C-3_D_), 76.1 (2 C, C-2_D_, C-5_B_), 75.6 (C-3_A_), 73.1 (C-4_D_), 71.8 (C-4_C_), 70.9 (C-3_B_), 70.6 (2 C, C-4_A_, C-4_B_), 70.0 (C-2_B_), 69.4 (C-5_D_), 68.8 (C-5_C_), 67.8 (C-5_A_), 67.1 (C-2_A_), 61.1 (C-6_B_), 60.8 (C-6_A_), 60.5 (C-6_C_), 56.4 (C-2_C_), 55.8 (O*C*H_3_), 23.2 (CO*C*H_3_), 16.4 (C*C*H_3_); ESI-MS: 820.3 [M+Na]^+^; Anal. Calcd. for C_33_H_51_NO_21_ (797.30): C, 49.68; H, 6.44%; found: 49.46; H, 6.69%.

### 4-Methoxyphenyl (α-l-rhamnopyranosyl)-(1→3)-(2-acetamido-2-deoxy-α-d-glucopyranosyl)-(1→3)-[β-d-mannopyranosyl-(1→4)]-α-d-galactopyranoside (2)

A solution of compound **18** (650.0 mg, 0.38 mmol) in 0.1 M CH_3_ONa in CH_3_OH (10 mL) was allowed to stir at room temperature for 1 h and neutralized with Amberlite IR 120 (H^+^) resin. The reaction mixture was filtered and concentrated. To a solution of the deacetylated product in anhydrous CH_2_Cl_2_ (5 mL) were added pyridine (2 mL) and triflic anhydride (200 µL, 1.19 mmol) and the reaction mixture was stirred at −10°C for 1 h. The solvents were removed under reduced pressure and triflate derivative was dissolved in HMPT-DMF (6 mL, 2∶1 v/v). To the solution of the triflate derivative was added NaN_3_ (500.0 mg, 7.69 mmol) and the reaction mixture was allowed to stir at 90 °C for 8 h. The reaction mixture was diluted with water and extracted with EtOAc (100 mL). The organic layer was successively washed with satd. NaHCO_3_ and water, dried (Na_2_SO_4_) and concentrated to give the crude product, which was passed through a short pad of SiO_2_. To a solution of the azido derivative in CH_3_OH (15 mL) was added 20% Pd(OH)_2_-C (200.0 mg) and the reaction mixture was stirred at room temperature for 24 h. The reaction mixture was filtered through a Celite® bed and evaporated to dryness. To a solution of the crude product in CH_3_OH (10 mL) was added acetic anhydride (2 mL) and the reaction mixture was allowed to stir at room temperature for 1 h. The solvents were removed under reduced pressure to give compound **2**, which was passed through a Sephadex® LH-20 column using CH_3_OH-H_2_O (4∶1) as eluant to give pure compound **2** (155.0 mg, 51%). White powder; [α]_D_^25^+32 (*c* 1.2, CH_3_OH); IR (KBr): 2932, 1757, 1452, 1388, 1233, 1113, 1086, 759, 697 cm^−1^; ^1^H NMR (CD_3_OD, 500 MHz): δ 7.10 (d, *J* = 9.0 Hz, 2 H, Ar-H), 6.80 (d, *J* = 9.0 Hz, 2 H, Ar-H), 5.38 (d, *J* = 3.5 Hz, 1 H, H-1_A_), 5.10 (d, *J* = 1.5 Hz, 1 H, H-1_C_), 4.90 (d, *J* = 1.5 Hz, 1 H, H-1_D_), 4.61 (br s, 1 H, H-1_B_), 4.43 (d, *J* = 2.5 Hz, 1 H, H-2_D_), 4.20 (dd, *J* = 10.5, 3.0 Hz, 1 H, H-3_A_), 4.09 (dd, *J* = 10.0, 3.5 Hz, 1 H, H-2_A_), 4.06-4.02 (m, 1 H, H-5_A_), 4.01-3.99 (m, 2 H, H-3_B_, H-5_D_), 3.98-3.95 (m, 1 H, H-3_D_), 3.94-3.92 (m, 3 H, H-3_C_, H-4_B_, H-6_aB_), 3.91-3.89 (m, 3 H, H-5_C_, H-6_aA_, H-6_aC_), 3.81 (dd, *J* = 9.0, 3.0 Hz, 1 H, H-2_C_), 3.77-3.74 (m, 2 H, H-4_C_, H-6_bA_), 3.73 (s, 3 H, OC*H*_3_), 3.65 (dd, *J* = 11.5, 8.0 Hz, 1 H, H-6_bC_), 3.54 (dd, *J* = 11.0, 6.0 Hz, 1 H, H-6_bB_), 3.50-3.46 (m, 2 H, H-2_B_, H-4_A_), 3.41 (t, *J* = 9.5 Hz each, 1 H, H-4_D_), 3.29-3.24 (m, 1 H, H-5_B_), 1.99 (s, 3 H, COC*H*_3_), 1.29 (d, *J* = 6.5 Hz, 3 H, CC*H*_3_); ^13^C NMR (CD_3_OD, 125 MHz): δ 169.8 (*C*OCH_3_), 156.7-115.5 (Ar-C), 102.6 (C-1_B_), 100.7 (C-1_A_), 99.6 (C-1_C_), 98.0 (C-1_D_), 78.2 (C-5_B_), 77.4 (C-3_A_), 76.3 (C-3_B_), 75.1 (C-2_B_), 74.6 (C-5_A_), 74.2 (C-4_D_), 73.9 (C-2_D_), 72.3 (C-2_C_), 72.2 (2 C, C-3_C_, C-5_D_), 72.1 (C-4_B_), 70.1 (C-5_C_), 69.0 (C-4_A_), 68.8 (C-2_A_), 68.2 (C-3_D_), 66.7 (C-4_C_), 63.2 (C-6_B_), 62.9 (C-6_A_), 61.3 (C-6_C_), 56.0 (O*C*H_3_), 22.4 (CO*C*H_3_), 18.1 (C*C*H_3_); ESI-MS: 820.3 [M+Na]^+^; Anal. Calcd. for C_33_H_51_NO_21_ (797.30): C, 49.68; H, 6.44%; found: 49.44; H, 6.70%.

## Supporting Information

Scheme S1 Reagents: (a) 0.1 M CH_3_ONa, CH_3_OH, room temperature, 3 h; (b) 2,2-dimethoxypropane, *p*-TsOH, DMF, room temperature, 12 h; (c) benzyl bromide, NaOH, DMF, room temperature, 4 h; (d) 80% aq. AcOH, 80 °C, 1.5 h; (e) Bu_2_SnO, MeOH, 70 °C, 3 h; (f) allyl bromide, DMF, TBAB, 60 °C, 8 h, 70% in six steps.
(TIF)

Scheme S2 Reagents: (a) TfOH, CH_2_Cl_2_, −25 °C, 1 h, 72%; (b) 0.1 M CH_3_ONa, CH_3_OH, room temperature, 2 h; (c) Dess-Martin Periodinane, CH_2_Cl_2_, room temperature, 1 h; (d) NaBH_4_, CH_3_OH, room temperature, 12 h; (e) acetic anhydride, pyridine, room temperature, 2 h, 76% in four steps; (f) PdCl_2_, CH_3_OH, room temperature, 1 h, 76% for **12** and 78% for **14**; (g) benzyl bromide, NaOH, Bu_4_NBr, THF, room temperature, 3 h, 90%.
(TIF)

Scheme S3 Reagents: (a) *N*-Iodosuccinimide, TfOH, CH_2_Cl_2_, MS 4 Å, −30 °C, 1 h, 77%.
(TIF)

Scheme S4 Reagents: (a) *N*-Iodosuccinimide, TfOH, CH_2_Cl_2_, MS 4 Å, −30 °C, 1 h, 72%; (b) NH_2_NH_2_·H_2_O, EtOH, 80 °C, 6 h; (c) acetic anhydride, pyridine, room temperature, 1 h; (d) H_2_, 20% Pd(OH)_2_-C, CH_3_OH, room temperature, 24 h; (e) 0.1 M CH_3_ONa, CH_3_OH, room temperature, 4 h, 57% in four steps.
(TIF)

Scheme S5 Reagents: (a) a) *N*-Iodosuccinimide, TfOH, CH_2_Cl_2_, MS 4 Å, −30 °C, 30 min, then compound **14** followed by NIS and TfOH, −30 °C, 30 min, 71%; (b) 0.1 M CH_3_ONa, CH_3_OH, room temperature, 1 h; (c) triflic anhydride, pyridine, CH_2_Cl_2_, −10 °C, 1 h; (d) NaN_3_, HMPT-DMF, 90 °C, 8 h; (e) H_2_, 20% Pd(OH)_2_-C, CH_3_OH, room temperature, 24 h; (f) acetic anhydride, CH_3_OH, room temperature, 1 h, 51% in four steps.
(TIF)
